# Mammographic calcifications association with risk of advanced breast cancer

**DOI:** 10.1007/s10549-025-07753-z

**Published:** 2025-06-17

**Authors:** Karla Kerlikowske, Linn Abraham, Brian L. Sprague, Olivia Sattayapiwat, Sarah J. Nyante, Jeffrey A. Tice, Diana L. Miglioretti

**Affiliations:** 1 Departments of Medicine and Epidemiology and Biostatistics, University of California, San Francisco, CA USA; 2 General Internal Medicine Section, Department of Veterans Affairs, University of California, San Francisco, CA USA; 3 Kaiser Permanente Washington Health Research Institute, Kaiser Permanente Washington, Seattle, WA USA; 4 Departments of Surgery and Radiology, University of Vermont, Burlington, VT USA; 5 Department of Public Health Sciences, University of California, Davis, CA USA; 6 Department of Radiology, University of North Carolina, Chapel Hill, NC USA; 7 Division of General Internal Medicine, Department of Medicine, University of California, San Francisco, CA USA; 8 General Internal Medicine Section, San Francisco Veterans Affairs Medical Center, 111A1, 4150 Clement Street, San Francisco, CA 94121 USA

**Keywords:** Advanced cancer, Mammographic calcification, Breast cancer risk

## Abstract

**Purpose:**

Mammographic calcifications on mammograms with a negative/benign assessment are associated with increased breast cancer risk. Associations with advanced breast cancer risk are unknown. We evaluated whether calcifications recorded on mammography reports are associated with advanced invasive breast cancer risk.

**Methods:**

We included 3,710,313 screening mammograms with a negative/benign final assessment performed on 991,991 women aged 40–74 in the Breast Cancer Surveillance Consortium associated with 7229 advanced cancers. We calculated cumulative 5-year advanced (prognostic pathologic stage ≥II) breast cancer risk and hazards ratios (HR) adjusted for clinical risk factors according to presence or absence of calcifications by menopausal status, dense (heterogeneously or extremely dense) vs. non-dense (almost entirely fatty or scattered fibroglandular density) breasts, body mass index (BMI) < 25 kg/m^2^ vs. ≥ 25 kg/m^2^.

**Results:**

Prevalence of calcifications was 6.1% among women who developed advanced breast cancer vs. 3.6% among others. Overall associations of advanced cancer with calcifications were similar for premenopausal (HR = 1.4; 95% CI 1.1–1.9) and postmenopausal (HR = 1.5; 95% CI 1.2–1.7) women. Compared to postmenopausal women with non-dense breasts and BMI < 25 kg/m^2^ without calcifications [cumulative 5-year advanced cancer incidence = 1.6 (95% CI 1.3–2.0) per 1000 women], postmenopausal women with dense breasts, BMI ≥ 25 kg/m^2^, and calcifications had 5.5-fold (95% CI 3.9–7.7) higher advanced cancer risk [cumulative 5-year advanced cancer incidence = 10.2; (95% CI 7.0–13.3) per 1000 women]. Results were similar for premenopausal women.

**Conclusion:**

Mammographic calcifications increase advanced cancer risk beyond having dense breasts and being overweight/obese. Future research should investigate strength of associations by type of calcification and incorporation of calcifications into advanced cancer risk models for improvement in model accuracy.

**Supplementary Information:**

The online version contains supplementary material available at 10.1007/s10549-025-07753-z.

## Introduction

Specific calcification patterns on imaging have long been recognized as a characteristic of benign or malignant lesions and recently have been associated with an increase in future breast cancer risk [1]. Multiple types of mammographic calcifications have been associated with breast cancer risk including calcifications identified by radiologists during clinical interpretation and subsequently associated with a false-positive mammogram [initial Breast Imaging Reporting and Data System (BI-RADS) score of 3, 4, 5, or 0 for which breast cancer was not detected after recall assessment] [1], microcalcifications (calcification clusters with a malignant morphology) associated with a negative/benign mammogram assessment [2, 3], and any type of mammographic calcification reported during clinical interpretation [4].

Mammographic calcifications have been associated with subsequent diagnosis of invasive breast cancer and ductal carcinoma in situ (DCIS) with a similar twofold increase in risk associations [3, 4]. In addition, mammographic calcifications have been included in risk prediction models and are associated with short- and long-term breast cancer risk among premenopausal and postmenopausal women [3–5]. Whether calcifications increase advanced invasive breast cancer risk is not known. Advanced breast cancer is a surrogate for breast cancer mortality [6] and has increasingly become an outcome examined when evaluating breast imaging effectiveness and risk [7–10]. For example, cancer detection with supplemental screening ultrasound has been shown to be higher among women with dense (heterogeneously or extremely dense) breasts and high advanced cancer risk compared to high invasive cancer risk [11].

The primary study goal was to evaluate whether mammographic calcifications noted by a radiologist in a mammography report with a BI-RADS initial or final assessment of negative or benign are a marker of advanced invasive breast cancer risk and whether associations between calcifications and advanced cancer risk vary by breast density, body mass index (BMI) and menopausal status using data from facilities in the Breast Cancer Surveillance Consortium (BCSC). As a secondary aim, we examined the association of mammographic calcifications with non-advanced invasive breast cancer.

Methods.

### Study setting and data sources

Data were from 113 facilities participating in one of four BCSC breast imaging registries that collected calcification data: San Francisco Mammography Registry, Carolina Mammography Registry, Vermont Breast Cancer Surveillance System, and Kaiser Permanente Washington system (https://www.bcsc-research.org/about/sites). We prospectively collected women’s characteristics and mammography information from radiology facilities. Breast cancer diagnoses and tumor characteristics were obtained by linking women to pathology databases; regional Surveillance, Epidemiology, and End Results programs; and state tumor registries. Deaths were obtained by linking to state death records. Registries and a central Statistical Coordinating Center (SCC) received Institutional Review Board approval for active or passive consenting processes or a waiver of consent to enroll participants, link data, and perform analyses. All procedures were Health Insurance Portability and Accountability Act compliant, and registries and the SCC received a Federal Certificate of Confidentiality and other protections for the identities of women, physicians, and facilities.

### Participants

This study included screening mammograms with a negative/benign initial or final assessment conducted from January 1996 through December 2019 among women aged 40–74 years. Screening mammograms were defined using the BCSC standard definition, which is based on a radiologist’s report of screening indication and excludes mammograms in women with a history of breast cancer or a prior mammogram within 9 months [12]. Only examinations with an initial assessment (based on screening views only) of BI-RADS 1 (negative), 2 (benign finding), or 0 (needs additional imaging) with a final assessment after any diagnostic work-up of BI-RADS 1 or 2 were included to avoid inclusion of cancers directly related with the calcification (*N* = 177546 excluded). Women were followed from 3 months after the mammogram to the earliest of the following: breast cancer diagnosis (advanced or non-advanced cancer, DCIS), death, disenrollment, end of complete cancer capture, or 5 years after the screen. Screening mammograms with less than 3 months of follow-up time (*N* = 108223) and examinations with cancers diagnosed within 3 months of screening (*N* = 16816) were excluded. Thus, we identified 3,710,313 screening mammograms with a negative/benign final assessment among 991,911 women (Supplemental Fig. 1).

### Measures, definitions, outcomes

We collected demographic and breast health history information from self-administered surveys at the time of screening and/or from electronic health records. Women self-reported race and ethnicity separately reported as the following categories: Hispanic/Latinx and non-Hispanic/Latinx African American/Black, Asian/Pacific Islander, White, Other/multiracial (non-Hispanic/Latinx Native American/Alaskan Native, or with two or more reported races, or other).

Radiologists (*N* = 932) categorized breast density during clinical interpretation using BI-RADS [13] breast density categories (Table 1) with a single radiologist providing an interpretation per exam. Mammographic calcifications were reported during clinical interpretation by radiologists and recorded in clinical electronic radiology systems.

**Table 1:** Characteristics of 3,710,313 screening mammograms among 991,911 women from 1996 to 2019 by cancer outcomes during 5 years of follow-up. Numbers in italics denote unknown values compared to known values which are not italized

	Total	No breast cancer		Advanced invasive breast cancer^a^		Non-advanced invasive breast cancer^b^	
	*N* (col %)^c^	*N* (col %)^c^	Row %	*N* (col %)^c^	Row %	*N* (col %)^c^	Row %
Screening examinations (*N*)	3,710,313	3,654,546		7229		36,637	
Age, years							
40–49	1,091,021 (29.4)	1,079,146 (29.5)	98.9	1856 (25.7)	0.17	7271 (19.8)	0.67
50–59	1,297,481 (35.0)	1,279,166 (35.0)	98.6	2603 (36.0)	0.20	11,648 (31.8)	0.90
60–69	986,718 (26.6)	968,046 (26.5)	98.1	2027 (28.0)	0.21	12,780 (34.9)	1.3
70–74	335,093 (9.0)	328,188 (9.0)	97.9	743 (10.3)	0.22	4938 (13.5)	1.5
Race/ethnicity							
Asian/Pacific Islander	328,054 (9.1)	323,482 (9.1)	98.6	447 (6.4)	0.14	2631 (7.3)	0.80
Black, non-Hispanic	336,843 (9.4)	332,205 (9.4)	98.6	1025 (14.6)	0.30	2664 (7.4)	0.79
Hispanic	125,053 (3.5)	123,674 (3.5)	98.9	210 (3.0)	0.17	829 (2.3)	0.66
White, non-Hispanic	2,743,616 (76.2)	2,700,469 (76.1)	98.4	5221 (74.3)	0.19	29,185 (81.2)	1.06
Other/multiracial	68,540 (1.9)	67,560 (1.9)	98.6	125 (1.8)	0.18	637 (1.8)	0.93
Unknown	*108,207 (2.9)*	*107,156 (2.9)*	*99.0*	*201 (2.8)*	*0.19*	*691 (1.9)*	*0.64*
Menopausal status							
Premenopausal	1,297,168 (35.0)	1,281,868 (35.1)	98.8	2332 (32.3)	0.18	9478 (25.9)	0.73
Postmenopausal^d^	2,413,145 (65.0)	2,372,678 (64.9)	98.3	4897 (67.7)	0.20	27,159 (74.1)	1.13
1 st degree family history of breast cancer^e^							
No	2,758,355 (84.1)	2,720,615 (84.3)	98.6	4819 (78.5)	0.17	24,665 (75.5)	0.89
Yes	520,402 (15.9)	508,556 (15.7)	97.7	1317 (21.5)	0.25	8017 (24.5)	1.54
Unknown	*431,556 (11.6)*	*425,375 (11.6)*	*98.6*	*1093 (15.1)*	*0.25*	*3955 (10.8)*	*0.92*
History of breast biopsy							
None (no prior biopsy)	2,966,815 (80.0)	2,927,847 (80.1)	98.7	5180 (71.7)	0.17	25,522 (69.7)	0.86
Prior biopsy, benign diagnosis unknown	540,743 (14.6)	529,041 (14.5)	97.8	1469 (20.3)	0.27	7744 (21.1)	1.43
Non-proliferative	144,717 (3.9)	141,465 (3.9)	97.8	409 (5.7)	0.28	2160 (5.9)	1.49
Proliferative without atypia	47,974 (1.3)	46,670 (1.3)	97.3	121 (1.7)	0.25	835 (2.3)	1.74
Proliferative with atypia	8625 (0.2)	8208 (0.2)	95.2	39 (0.5)	0.45	283 (0.8)	3.28
LCIS	1439 (0.0)	1315 (0.0)	91.4	11 (0.2)	0.76	93 (0.3)	6.46
BI-RADS breast density^f^							
Almost entirely fat	317,430 (9.5)	314,713 (9.5)	99.1	214 (3.3)	0.07	1898 (5.8)	0.60
Scattered fibroglandular density	1,501,985 (44.8)	1,481,769 (44.9)	98.7	2390 (36.4)	0.16	13,704 (41.5)	0.91
Heterogeneously dense	1,275,470 (38.0)	1,253,179 (37.9)	98.3	3251 (49.5)	0.25	14,423 (43.7)	1.13
Extremely dense	258,344 (7.7)	253,529 (7.7)	98.1	716 (10.9)	0.28	2966 (9.0)	1.15
Unknown	*357,084 (9.6)*	*351,356 (9.6)*	*98.4*	*658 (9.1)*	*0.18*	*3646 (10.0)*	*1.02*
Body mass index, kg/m^2^							
Underweight (< 18.5)	31,300 (1.5)	30,913 (1.5)	98.8	38 (1.1)	0.12	226 (1.1)	0.72
Normal (18.5–24.9)	862,718 (41.9)	850,460 (41.9)	98.6	1215 (35.3)	0.14	8051 (38.9)	0.93
Overweight (25.0–29.9)	594,887 (28.9)	585,310 (28.8)	98.4	1082 (31.4)	0.18	6464 (31.2)	1.09
Obese I (30.0–34.9)	320,535 (15.6)	315,449 (15.5)	98.4	634 (18.4)	0.20	3434 (16.6)	1.07
Obese II/III (≥ 35.0)	250,905 (12.2)	247,223 (12.2)	98.5	472 (13.7)	0.19	2515 (12.2)	1.00
Unknown	*1,649,968 (44.5)*	*1,625,191 (44.5)*	*98.5*	*3788 (52.4)*	*0.23*	*15,947 (43.5)*	*0.97*
Time since last mammogram							
1 year	2,156,019 (63.0)	2,118,848 (62.9)	98.3	4344 (64.5)	0.20	24,684 (71.2)	1.14
2 years	722,070 (21.1)	712,216 (21.1)	98.6	1395 (20.7)	0.19	6429 (18.6)	0.89
3 + years	416,601 (12.2)	411,897 (12.2)	98.9	779 (11.6)	0.19	2972 (8.6)	0.71
First	127,667 (3.7)	126,711 (3.8)	99.3	215 (3.2)	0.17	563 (1.6)	0.44
Unknown	*287,956 (7.8)*	*284,874 (7.8)*	*98.9*	*496 (6.9)*	*0.17*	*1989 (5.4)*	*0.69*
Mammography calcifications							
No^g^	3,573,740 (96.3)	3,521,321 (96.4)	98.5	6787 (93.9)	0.19	34,365 (93.8)	0.96
Yes	136,573 (3.7)	133,225 (3.6)	97.6	442 (6.1)	0.32	2272 (6.2)	1.66
Initial BI-RADS assessment							
1 (negative)	2,616,848 (70.5)	2,582,033 (70.7)	98.7	4396 (60.8)	0.17	22,970 (62.7)	0.88
2 (benign finding)	898,229 (24.2)	881,214 (24.1)	98.1	2303 (31.9)	0.26	11,072 (30.2)	1.23
0 (needs additional imaging)	195,236 (5.3)	191,299 (5.2)	98.0	530 (7.3)	0.27	2595 (7.1)	1.33
Final BI-RADS assessment							
1 (negative)	2,701,503 (72.8)	2,665,302 (72.9)	98.7	4565 (63.1)	0.17	23,912 (65.3)	0.89
2 (benign finding)	1,008,810 (27.2)	989,244 (27.1)	98.1	2664 (36.9)	0.26	12,725 (34.7)	1.26

^a^Invasive cancer American Joint Committee on Cancer (AJCC) 8th edition prognostic pathologic stage II or higher

^b^Invasive cancer AJCC 8th edition prognostic pathologic stage I

^c^Column percentages for non-missing categories are based on exams not missing characteristic

^d^Current hormone therapy use, natural menopause, ovaries removed, age 60 or older, last period ≥ 365 days prior

^e^Defined as first-degree relative (mother, sister, or daughter) with breast cancer

^f^Breast imaging reporting and data system (BI-RADS)

^g^Includes missing

Postmenopausal women were those with both ovaries removed, whose periods had stopped naturally, who currently used postmenopausal hormone therapy, or who were age 60 or older [14]. Premenopausal women reported a period within the last 180 days or did not meet one of the postmenopausal criteria and used birth control hormones. If a woman did not meet any of these criteria, then age at screen was used to classify a woman as postmenopausal (age ≥ 52) or premenopausal (age < 52) [14–17]. BMI was categorized as < 18.5 kg/m^2^ = underweight, 18.5–24.9 kg/m^2^ = normal weight, 25.0–29.9 kg/m^2^ = overweight, 30.0–34.9 kg/m^2^ = obese I, and ≥ 35.0 kg/m^2^ = obese II/III [18].

The primary outcome was diagnosis of advanced invasive breast cancer, defined as prognostic pathologic stage II or higher [6]. We classified American Joint Committee on Cancer 8th edition prognostic pathologic stage [19] using anatomic staging elements, tumor grade, and estrogen, progesterone, and human epidermal growth factor receptor status. If prognostic stage variables were missing (34%), we used anatomic stage IIB or higher (27%) or summary stage or other information (6%) to classify as advanced cancer [6]. Advanced cancer status was imputed for the remaining 1.5% of screens using an imputation model including tumor characteristics. The secondary outcome was prognostic pathologic stage I (non-advanced cancer).

### Statistical approach

Patient characteristics were summarized across advanced and non-advanced breast cancer stage, and the presence of calcifications (no, yes). Multiple imputation using fully conditional specification methods [20] was used to impute 108,207 (2.9%) missing values of race/ethnicity, 287,956 (7.8%) missing values of time since last mammogram, 357,084 (9.6%) missing values of breast density, 431,556 (11.6%) missing values of first-degree family history of breast cancer, and 1,649,968 (44.5%) missing values in BMI in 45 imputed datasets [21]. Imputation models included the characteristics, time to event, and the Nelson-Aalen estimator [20], weighted by the inverse number of mammograms per woman [22]. Prevalence of calcifications and variances were estimated for each imputed dataset and combination of menopausal status, breast density, and BMI. Prevalences were averaged across the imputed datasets, and Rubin’s Rule [23] was used to compute pooled standard errors to estimate Wald-type 95% confidence intervals.

To obtain unadjusted cumulative incidence functions (CIF) and standard errors, one observation per woman was randomly chosen within each imputed dataset. The CIF for each tumor type and imputed dataset was estimated using SAS PROC LIFETEST, subdivided by presence of calcifications, menopausal status, breast density and BMI category; the other tumor type and DCIS were considered competing risks. Five-year risks were averaged across the imputed datasets, and Rubin’s Rule [23] was used to compute pooled standard errors and estimate Wald-type 95% confidence intervals.

To estimate the association between advanced and non-advanced cancer risk and presence of calcifications, we estimated adjusted hazard ratios (HRs) based on Fine and Gray subdistribution hazard models accounting for competing risks of the other tumor outcomes. Models included interactions between presence of calcifications and menopausal status, BMI, breast density, and initial assessment (BI-RADS 1,2 vs BI-RADS 0) depending on the HRs being estimated. All models adjusted for age at screen (quadratic), race/ethnicity, family history of breast cancer, history of benign biopsy, and time since last mammogram, and were stratified by BCSC registry. A robust sandwich variance estimator and inverse-weighting by the number of screening mammograms per woman were used to account for multiple screens per woman [22, 24]. Results from the 45 imputed datasets were combined using PROC MIANALYZE in SAS.

To evaluate whether the association between presence of calcifications and advanced cancer risk varied over time, we refit the survival model with time-varying indicators of the presence of calcifications within three-time intervals; < 1 year, 1–3 years, and 3–5 years.

Data were analyzed using R version 4.0.4 (R Foundation for Statistical Computing, Vienna, Austria) and SAS version 9.4 (SAS Institute, Cary, NC). Two-sided alpha = 0.05 was used to determine statistical significance.

## Results

We included 3,710,313 screening mammograms performed on 991,991 women aged 40–74 years associated with 7,229 diagnoses of advanced cancer, 36,637 non-advanced invasive cancer, and 11,901 DCIS during the 5-year follow-up period. Mammograms associated with an advanced or non-advanced invasive breast cancer during follow-up occurred in women who were older, had a first-degree family history of breast cancer, prior history of benign breast biopsy, dense breasts, and were overweight or obese compared to women without breast cancer (Table 1). Women with advanced cancer were more likely to be Black, obese, or have dense breasts compared to women without breast cancer or non-advanced breast cancer.

**Fig. 1:**
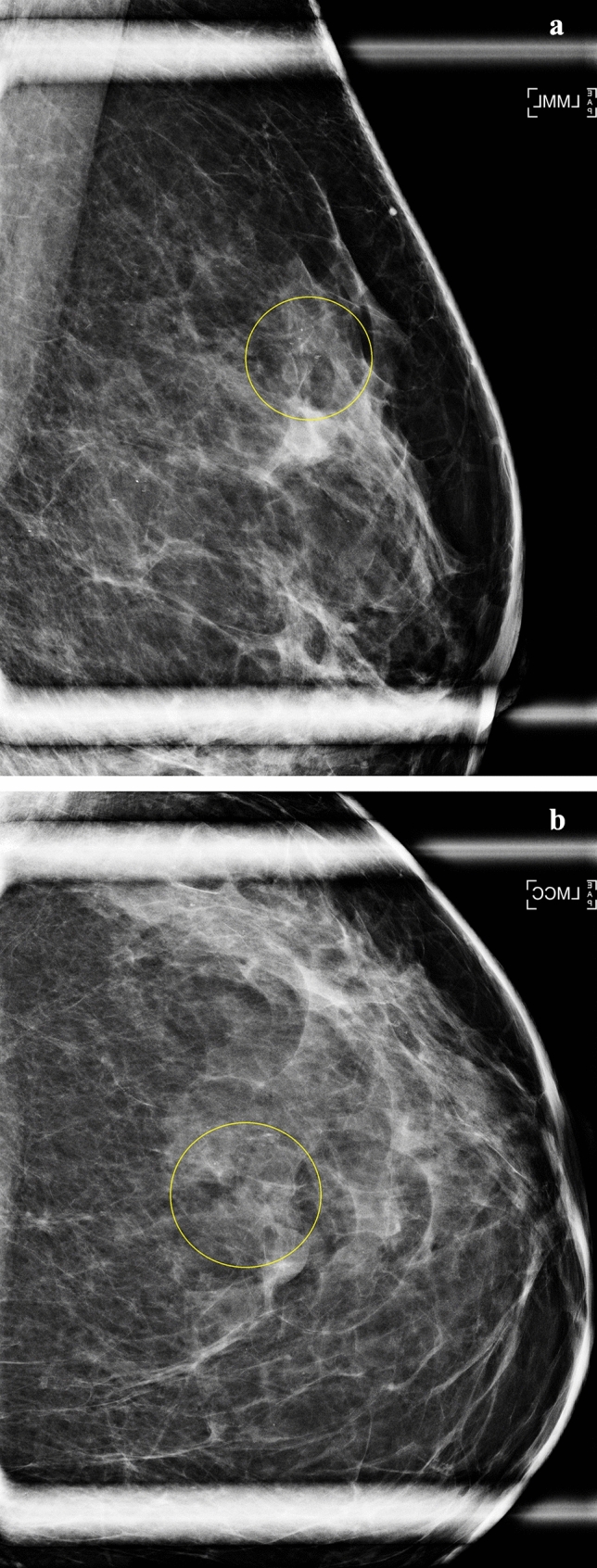
52-year-old woman recalled from screening mammography for further evaluation of left breast calcifications in the upper outer quadrant with final assessment of BI-RADS 2

Of the 3.7% of examinations with calcifications, 88.3% had an initial negative/benign assessment and 11.7% had an initial BI-RADS 0/final negative/benign assessment (Tables 1 and 2). The prevalence of calcifications ranged from 6.1% to 6.2% among women who developed a subsequent breast cancer diagnosis vs. 3.6% among others (Table 1). An example of a mammogram with a BI-RADS 2 assessment with calcifications is shown in Fig. 1) Women with calcifications tended to be White, postmenopausal, and have a first-degree family history of breast cancer or history of breast biopsy compared to women without calcifications and have an initial or final assessment of BI-RADS 2 (Table 2). Prevalence of calcifications increased with increasing age, breast density, and BMI. Postmenopausal women with dense breasts and BMI ≥ 25 kg/m^2^ had the highest prevalence of calcifications at 6.0% (Supplemental Table 1).

**Table 2:** Characteristics of 3,710,313 screening mammograms among 991,911 women from 1996 to 2019 by presence of calcifications

	Without calcifications^a^		With calcifications	
	Col %^b^	Row %	Col %^b^	Row %
Screening examinations (N)	3,573,740		136,573	
Age, years				
40–49	1,066,704 (29.8)	97.8	24,317 (17.8)	2.2
50–59	1,251,387 (35.0)	96.5	46,094 (33.8)	3.6
60–69	939,315 (26.3)	95.2	47,403 (34.7)	4.8
70–74	316,334 (8.9)	94.4	18,759 (13.7)	5.6
Race/ethnicity				
Asian/Pacific Islander	321,814 (9.3)	98.1	6240 (4.6)	1.9
Black, non-Hispanic	327,225 (9.4)	97.1	9618 (7.1)	2.9
Hispanic	120,765 (3.5)	96.6	4288 (3.2)	3.4
White, non-Hispanic	2,631,896 (75.9)	95.9	111,720 (82.6)	4.1
Other/multiracial	65,083 (1.9)	95.0	3457 (2.6)	5.0
Unknown	*106,957 (3.0)*	*98.8*	*1250 (0.9)*	*1.2*
Menopausal status				
Premenopausal	1,265,267 (35.4)	97.5	31,901 (23.4)	2.5
Postmenopausal^c^	2,308,473 (64.6)	95.7	104,672 (76.6)	4.3
1 st degree family history of breast cancer^d^				
No	2,658,031 (84.3)	96.4	100,324 (80.4)	3.6
Yes	495,940 (15.7)	95.3	24,462 (19.6)	4.7
Unknown	*419,769 (11.7)*	*97.3*	*11,787 (8.6)*	*2.7*
History of breast biopsy				
None (no prior biopsy), unknown	2,872,013 (80.4)	96.8	94,802 (69.4)	3.2
Prior biopsy, benign diagnosis unknown	511,668 (14.3)	94.6	29,075 (21.3)	5.4
Non-proliferative	135,581 (3.8)	93.7	9136 (6.7)	6.3
Proliferative without atypia	45,045 (1.3)	93.9	2929 (2.1)	6.1
Proliferative with atypia	8083 (0.2)	93.7	542 (0.4)	6.3
LCIS	1350 (0.0)	93.8	89 (0.1)	6.2
BI-RADS breast density^e^				
Almost entirely fat	307,720 (9.6)	96.9	9710 (7.2)	3.1
Scattered fibroglandular density	1,448,668 (45.0)	96.5	53,317 (39.5)	3.6
Heterogeneously dense	1,218,303 (37.9)	95.5	57,167 (42.4)	4.5
Extremely dense	243,643 (7.6)	94.3	14,701 (10.9)	5.7
Unknown	*355,406 (9.9)*	*99.5*	*1678 (1.2)*	*0.5*
Body mass index, kg/m^2^				
Underweight (< 18.5)	29,933 (1.5)	95.6	1367 (1.4)	4.4
Normal (18.5–24.9)	826,589 (42.1)	95.8	36,129 (36.6)	4.2
Overweight (25.0–29.9)	566,641 (28.9)	95.3	28,246 (28.6)	4.8
Obese I (30.0–34.9)	303,116 (15.5)	94.6	17,419 (17.6)	5.4
Obese II/III (≥ 35.0)	235,319 (12.0)	93.8	15,586 (15.8)	6.2
Unknown	*1,612,142 (45.1)*	*97.7*	*37,826 (27.7)*	*2.3*
Time since last mammogram				
1 year	2,078,052 (63.1)	96.4	77,967 (60.8)	3.6
2 years	689,885 (20.9)	95.5	32,185 (25.1)	4.5
3 + years	401,674 (12.2)	96.4	14,927 (11.6)	3.6
First mammogram	124,548 (3.8)	97.6	3119 (2.4)	2.4
Unknown	*279,581 (7.8)*	*97.1*	*8375 (6.1)*	*2.9*
Initial BI-RADS assessment				
1 (negative)	2,611,136 (73.1)	99.8	5712 (4.2)	0.2
2 (benign finding)	783,327 (21.9)	87.2	114,902 (84.1)	12.8
0 (needs additional imaging)	179,277 (5.0)	91.8	15,959 (11.7)	8.2
Final BI-RADS assessment				
1	2,693,214 (75.4)	99.7	8289 (6.1)	0.31
2	880,526 (24.6)	87.3	128,284 (93.9)	12.7

^a^Includes missing

^b^Column percentages are based on non-missing values

^c^Current hormone therapy use, natural menopause, ovaries removed, age 60 or older, last period ≥ 365 days prior

^d^Defined as first-degree relative (mother, sister, or daughter) with breast cancer

^e^Breast imaging reporting and data system (BI-RADS)

In multivariable analyses, overall associations with calcifications were similar for advanced cancer and non-advanced cancer, but associations with BMI and breast density were stronger for advanced than non-advanced cancer (Supplemental Table 2). The overall association of advanced cancer with calcifications were similar for premenopausal (HR = 1.4, 95% CI 1.1–1.9) and for postmenopausal women (HR = 1.5, 95% CI 1.2–1.7) adjusting for breast density and BMI (Table 3). Statistically significant associations between the presence of calcifications and advanced breast cancer were found among premenopausal obese women (HR = 1.7; 95% CI 1.1–2.9) and postmenopausal overweight (HR = 1.3; 95% CI 1.0–1.8) and obese (HR = 1.7; 95% CI 1.3–2.1) women compared to women without calcifications in the corresponding menopausal and BMI categories (Table 3). Associations between the presence of calcifications and non-advanced invasive cancer were similar for premenopausal and postmenopausal women regardless of BMI.

**Table 3:** Unadjusted cumulative 5-year risk and multivariable hazard ratios for advanced and non-advanced invasive breast cancer for mammograms with vs. without mammographic calcifications by menopausal status and body mass index

Clinical and imaging risk factors	Advanced cancer^a^		Non-advanced invasive cancer^b^	
	Unadjusted 5-year risk (95% CI)^c^	HR (95% CI)	Unadjusted 5-year risk (95% CI)^c^	HR (95% CI)
No mammography calcifications	3.1 (3.0, 3.3)	Ref.	13.4 (13.1, 13.6)	Ref.
Mammography calcifications^d^	5.6 (4.6, 6.6)	1.5 (1.3,1.7)	25.6 (23.4, 27.9)	1.4 (1.4,1.5)
Premenopausal women^e^				
No mammography calcifications	2.9 (2.7, 3.1)	Ref.	10.0 (9.7, 10.4)	Ref.
Mammography calcifications	5.1 (3.4, 6.7)	1.4 (1.1,1.9)	18.7 (15.2, 22.1)	1.5 (1.3,1.7)
BMI < 25 kg/m^2^, no mammography calcifications	2.6 (2.3, 2.9)	Ref.	11.5 (10.9, 12.1)	Ref.
BMI < 25 kg/m^2^, mammography calcifications	4.0 (1.7, 6.4)	1.3 (0.9,2.0)	21.1 (15.8, 26.4)	1.5 (1.3,1.8)
BMI 25–29 kg/m^2^, no mammography calcifications	3.2 (2.7, 3.7)	Ref.	9.7 (8.9, 10.5)	Ref.
BMI 25–29 kg/m^2^, mammography calcifications	5.0 (1.2, 8.9)	1.2 (0.7,2.3)	16.7 (9.3, 24.1)	1.4 (1.0,1.8)
BMI ≥ 30 kg/m^2^, no mammography calcifications	3.2 (2.8, 3.6)	Ref.	8.0 (7.3, 8.6)	Ref.
BMI ≥ 30 kg/m^2^, mammography calcifications	7.0 (3.1, 11.0)	1.7 (1.1,2.9)	16.0 (9.5, 22.4)	1.5 (1.1,2.0)
Postmenopausal women^e^				
No mammography calcifications	3.3 (3.1, 3.5)	Ref.	15.9 (15.5, 16.3)	Ref.
Mammography calcifications	5.9 (4.6, 7.1)	1.5 (1.2,1.7)	28.7 (25.9, 31.4)	1.4 (1.3,1.6)
BMI < 25 kg/m^2^, no mammography calcifications	2.6 (2.3, 2.9)	Ref.	15.8 (15.1, 16.5)	Ref.
BMI < 25 kg/m^2^, mammography calcifications	4.0 (2.1, 6.0)	1.3 (0.9,1.8)	29.1 (24.3, 33.9)	1.5 (1.4,1.7)
BMI 25–29 kg/m^2^, no mammography calcifications	3.5 (3.1, 3.9)	Ref.	16.7 (15.9, 17.5)	Ref.
BMI 25–29 kg/m^2^, mammography calcifications	5.7 (3.3, 8.2)	1.3 (1.0,1.8)	29.3 (24.0, 34.6)	1.4 (1.2,1.6)
BMI ≥ 30 kg/m^2^, no mammography calcifications	3.9 (3.5, 4.3)	Ref.	15.4 (14.6, 16.1)	Ref.
BMI ≥ 30 kg/m^2^, mammography calcifications	7.8 (5.3, 10.2)	1.7 (1.3,2.1)	27.8 (23.0, 32.7)	1.4 (1.2,1.6)

^a^Invasive cancer American Joint Committee on Cancer (AJCC) 8th edition prognostic pathologic stage II or higher

^b^Invasive cancer AJCC 8th edition prognostic pathologic stage I

^c^Unadjusted 5-year risk was estimated for each outcome per 1000 women, treating the other tumor type and DCIS as competing risks

^d^Hazard ratios for presence of calcifications vs absence of calcifications are based on models that included presence of calcifications, adjusted for menopausal status, 3-category BMI, their interaction, age (quadratic), race/ethnicity, 1 st degree breast cancer family history, history of benign biopsy, breast density, time since last mammogram, and stratified by BCSC registry. One model was fit for each outcome, while treating the other tumor type and DCIS as competing risks

^e^Hazard ratios for presence of calcifications vs absence of calcifications by menopausal status were based on models including presence of calcifications, menopause, 3-category BMI, the interaction between menopause and calcifications, and adjusted for age (quadratic), race/ethnicity, 1 st degree breast cancer family history, history of benign biopsy, breast density, time since last mammogram, and stratified by BCSC registry. Hazard ratios by BMI and menopausal status were based on similar models that additionally included all interactions between presence of calcifications, menopause, and BMI. One model was fit for each outcome, while treating the other tumor type and DCIS as competing risks

**Fig. 2:**
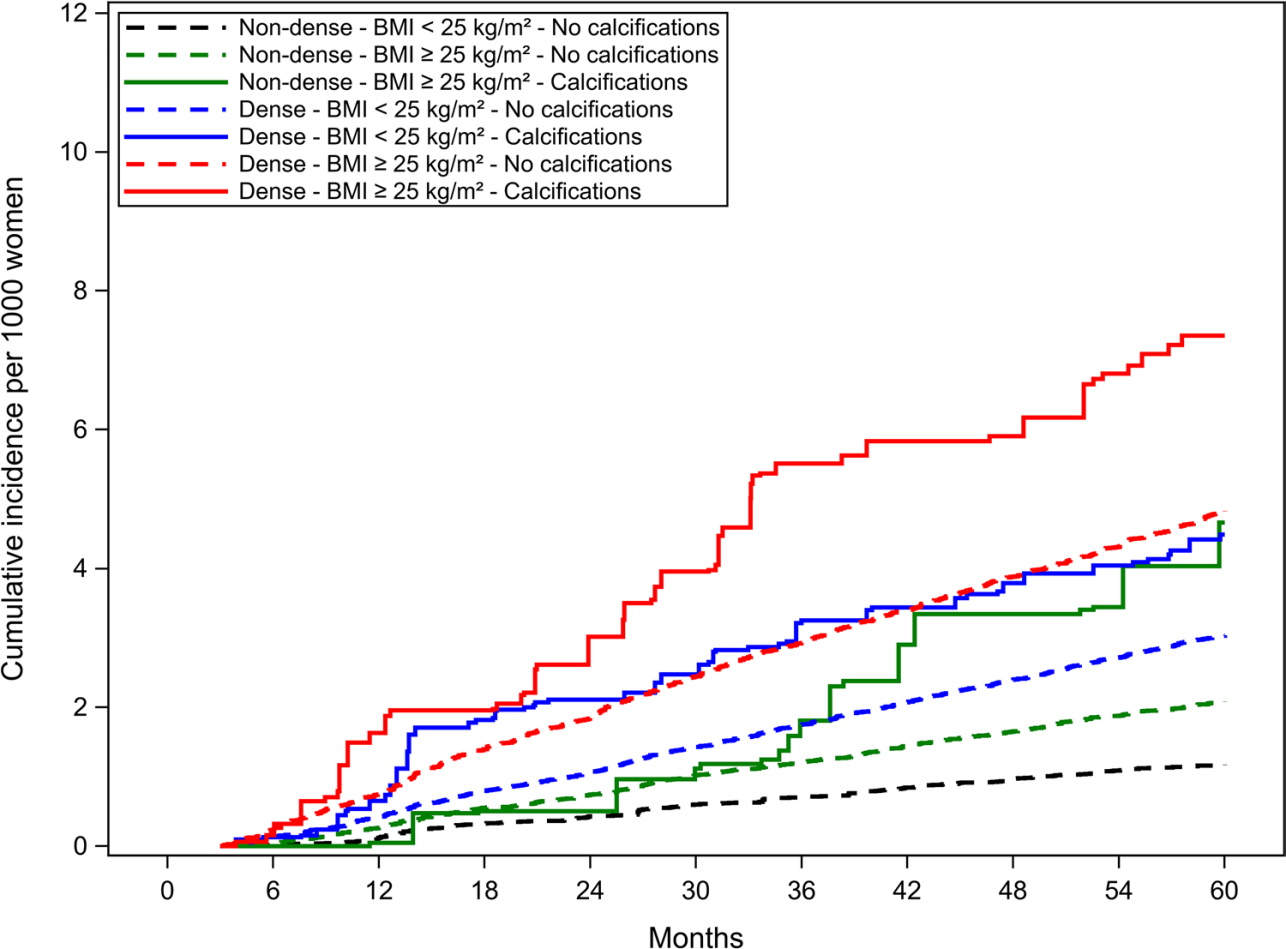
Unadjusted cumulative incidence function for advanced invasive cancer in pre-menopausal women

**Fig. 3:**
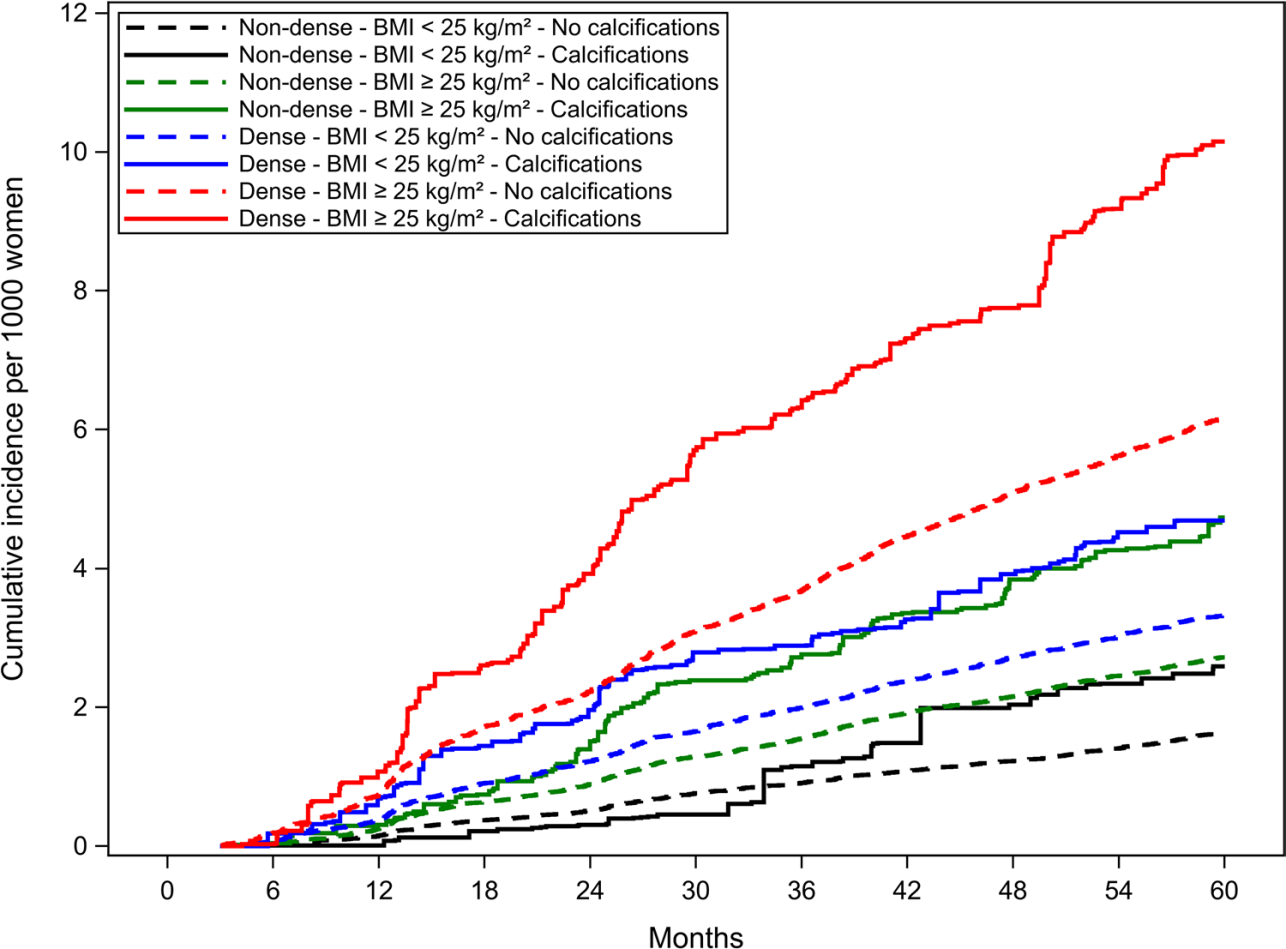
Unadjusted cumulative incidence function for advanced invasive cancer in post-menopausal women

The cumulative incidence of advanced cancer overall was lower for premenopausal (Fig. 2) than postmenopausal women (Fig. 3) and higher when calcifications were present for all breast density and BMI combinations. Postmenopausal and premenopausal women with calcifications, dense breasts and BMI ≥ 25 kg/m^2^ had the highest cumulative incidence of advanced breast cancer.

Compared to women with BMI < 25 kg/m^2^ and non-dense (almost entirely fatty or scattered fibroglandular density) breasts without calcifications, advanced cancer risk was higher for women with calcifications and dense breasts or BMI ≥ 25 kg/m^2^: premenopausal women with calcifications, dense breasts and BMI < 25 kg/m^2^ (HR = 3.7; 95% CI 2.0–6.7), or non-dense breasts and BMI ≥ 25 kg/m^2^ (HR = 3.2; 95% CI 1.3–7.5); postmenopausal women with calcifications, dense breasts and with BMI < 25 kg/m^2^ (HR = 2.6; 95% CI 1.6–4.2), or non-dense breasts and BMI ≥ 25 kg/m^2^ and (HR = 2.6; 95% CI 1.7–3.8) (Table 4).

**Table 4:** Unadjusted cumulative 5-year risk for advanced and non-advanced breast cancer and hazard ratios by mammographic calcifications, menopausal status, breast density and BMI

	Advanced cancer^a^		Non-advanced invasive cancer^b^	
Risk factors	Unadjusted 5-year risk (95% CI)^c^	HR (95% CI)^d^	Unadjusted 5-year risk (95% CI)^c^	HR (95% CI)^d^
Premenopausal, non-dense				
BMI < 25 kg/m^2^, no calcifications	1.2 (0.8, 1.6)	Ref.	8.3 (7.2, 9.3)	Ref.
BMI < 25 kg/m^2^, calcifications	–^e^	–^e^	16.5 (4.2, 28.7)	1.7 (1.0,2.9)
BMI ≥ 25 kg/m^2^, no calcifications	2.1 (1.8, 2.4)	1.7 (1.2, 2.4)	6.9 (6.3, 7.4)	0.9 (0.8,1.0)
BMI ≥ 25 kg/m^2^, calcifications	4.7 (1.3, 8.0)	3.2 (1.3, 7.5)	11.7 (5.6, 17.7)	1.3 (0.9,1.8)
Premenopausal, dense				
BMI < 25 kg/m^2^, no calcifications	3.0 (2.7, 3.4)	2.6 (1.9, 3.7)	12.5 (11.8, 13.2)	1.6 (1.4,1.7)
BMI < 25 kg/m^2^, calcifications	4.5 (1.9, 7.1)	3.7 (2.0, 6.7)	21.8 (16.0, 27.6)	2.3 (1.9,2.9)
BMI ≥ 25 kg/m^2^, no calcifications	4.8 (4.3, 5.4)	3.9 (2.7, 5.5)	11.6 (10.7, 12.6)	1.5 (1.3,1.7)
BMI ≥ 25 kg/m^2^, calcifications	7.4 (3.3, 11.4)	5.4 (2.8, 10.3)	20.3 (13.2, 27.4)	2.1 (1.7,2.8)
Postmenopausal, non-dense				
BMI < 25 kg/m^2^, no calcifications	1.6 (1.3, 2.0)	Ref.	13.2 (12.2, 14.2)	Ref.
BMI < 25 kg/m^2^, calcifications	2.6 (0, 5.6)	1.4 (0.6, 3.4)	25.7 (16.8, 34.6)	1.7 (1.4,2.1)
BMI ≥ 25 kg/m^2^, no calcifications	2.7 (2.5, 3.0)	1.6 (1.3, 1.9)	14.0 (13.4, 14.6)	1.1 (1.1,1.2)
BMI ≥ 25 kg/m^2^, calcifications	4.7 (3.0, 6.5)	2.6 (1.7, 3.8)	24.6 (20.4, 28.7)	1.7 (1.5,1.9)
Postmenopausal, dense				
BMI < 25 kg/m^2^, no calcifications	3.3 (2.9, 3.8)	2.0 (1.6, 2.5)	17.8 (16.8, 18.8)	1.4 (1.3,1.5)
BMI < 25 kg/m^2^, calcifications	4.7 (2.2, 7.2)	2.6 (1.6, 4.2)	30.7 (24.8, 36.7)	2.1 (1.8,2.4)
BMI ≥ 25 kg/m^2^, no calcifications	6.2 (5.5, 6.9)	3.5 (2.9, 4.4)	21.1 (19.9, 22.3)	1.7 (1.6,1.8)
BMI ≥ 25 kg/m^2^, calcifications	10.2 (7.0, 13.3)	5.5 (3.9, 7.7)	34.6 (28.7, 40.6)	2.4 (2.1,2.7)

^a^Invasive cancer American Joint Committee on Cancer (AJCC) 8th edition prognostic pathologic stage II or higher

^b^Invasive cancer AJCC 8th edition prognostic pathologic stage I

^c^Unadjusted 5-year risk was estimated for each outcome per 1000 women, treating the other tumor type and DCIS as competing risks

^d^Models include menopausal status, binary density, binary BMI, presence of calcifications, and all interactions; adjusted for age and its quadratic term, race/ethnicity, 1 st degree breast cancer family history, history of benign biopsy, and time since last mammogram, and stratified by BCSC registry. Results are based on a parameterization where the reference group low density, BMI < 25 kg/m^2^, no calcifications, within each menopausal status. One model was fit for each outcome, while treating the other tumor type and DCIS as competing risks

^e^Estimate not reported since less than 10 outcomes in group; Non-dense (almost entirely fatty and scattered fibroglandular density); Dense (heterogeneously and extremely dense breast density); body mass index (BMI)

Among pre- and post-menopausal women, advanced cancer risk was highest among women with dense breasts, BMI ≥ 25 kg/m^2^, and calcifications. Compared to premenopausal women without dense breasts, BMI < 25 kg/m^2^, and without calcifications [cumulative 5-year advanced cancer incidence = 1.2 (95% CI 0.8–1.6) per 1000 women], premenopausal women with dense breasts, BMI ≥ 25 kg/m^2^, and calcifications had 5.4-fold (95% CI 2.8–10.3) higher advanced cancer risk [cumulative 5-year advanced cancer incidence = 7.4; (95% CI 3.3–11.4) per 1000 women] (Table 4). Compared to postmenopausal women without dense breasts, BMI < 25 kg/m^2^, and without calcifications [cumulative 5-year advanced cancer incidence 1.6; (95% CI 1.3–2.0) per 1000 women], postmenopausal women with dense breasts, BMI ≥ 25 mg/m^2^, and calcifications had 5.5-fold (95% CI 3.9–7.7) higher advanced cancer risk [cumulative 5-year advanced cancer incidence = 10.2 (95% CI 7.0–13.3) per 1000 women] (Table 4).

Cumulative 5-year non-advanced invasive cancer incidence was increased when calcifications were present among pre-menopausal and postmenopausal women with dense breasts, but risks were similar within calcification and breast density strata for those with BMI < 25 vs. BMI ≥ 25 kg/m^2^ (Table 4; Supplemental Figs. 2 and 3).

Associations with calcifications were similar following an initial BI-RADS assessment of 1 or 2 and BI-RADS 0 (final assessment BI-RADS assessment of 1 or 2) for advanced cancer and non-advanced cancer outcomes: Advanced cancer HR = 1.4 (95% CI 1.2–1.7) and HR = 1.4 (95% CI 1.0–2.1), respectively; Non-advanced invasive cancer HR = 1.4 (95% CI 1.3–1.5) and HR = 1.3 (95% CI 1.1–1.5), respectively.

Advanced cancer risk associated with the presence of calcifications did not vary significantly across the time intervals examined within the follow-up period (Supplemental Table 3).

## Discussion

This study examined whether calcifications reported by radiologists during clinical mammography interpretation were associated with advanced invasive breast cancer risk. Results from a large diverse U.S. screening mammography population showed mammographic calcifications were associated with increased advanced cancer risk, with the highest risks observed among premenopausal and postmenopausal women with dense breasts and BMI ≥ 25 kg/m^2^.

This is the first study to examine the association of mammographic calcifications with advanced invasive breast cancer and non-advanced invasive breast cancer separately and demonstrates that when combined with dense breasts and/or BMI ≥ 25 kg/m^2^ the association with advanced cancer differs from non-advanced breast cancer. Mammographic calcifications were associated with higher advanced cancer risk beyond having dense breasts and being overweight/obese whereas mammographic calcifications were associated with higher non-advanced cancer risk when combined with dense breasts regardless of BMI. Prior studies have examined the association of radiologists’ identified mammographic typically benign or suspicious morphology calcifications or only microcalcifications with risk of breast cancer (invasive combined with DCIS) and found a twofold increase in risk when mammographic calcifications were present compared with HR of 1.3–1.7 association with advanced cancer in our study [1, 2, 4]. This association appears robust and has been found in populations where prevalent cancers [4] and cancers diagnosed within the first 3 months of screening [2] were excluded from the study population and where only mammograms with a negative screening examination were examined [2]. Microcalcification clusters identified on negative screening mammograms using artificial intelligence (AI) also have been associated with the combined outcome of invasive cancer and DCIS [3, 5].

Dense breasts and obesity have both been associated with increased risk of breast cancer overall and of advanced breast cancer following routine mammography screening [7, 9, 25]. One study reported that mammographic microcalcifications are independently associated with breast cancer risk and the risk increased when combined with breast density [2]. We also show mammographic calcifications combined with dense breasts increased risk more than calcifications alone for advanced cancer and non-advanced invasive cancer for premenopausal and postmenopausal women. We extend the literature by showing overweight or obese women with mammographic calcifications have a higher advanced cancer risk than those with mammographic calcifications alone and, when combined with dense breasts have the highest advanced breast cancer risk.

Cancer genesis may depend on a chronic inflammatory state [26] and obesity, calcifications, and breast density have all been linked to an inflammatory environment in the breast [26]. Obesity is thought to increase breast cancer risk through local inflammation [27–29]. In support of this hypothesis, regular use of nonsteroidal anti-inflammatory medications has been shown to be associated with decreased breast cancer risk among overweight women [30]. In addition, decreased breast cancer risk has been associated with weight reduction through diet, exercise, and bariatric surgery [31–35]. An inflammatory profile of the breast tissue may contribute to breast tissue microcalcification development and breast carcinogenesis [36]. High mammographic breast density is characterized by high proportions of stroma containing fibroblasts, collagen and immune cells suggesting a pro-tumor inflammatory microenvironment [37]. Thus, women who are obese, have dense breasts, and mammographic calcifications may have a high local inflammatory state that promotes tumorigenesis.

An actionable risk model for advanced breast cancer was developed to predict cumulative six-year risk of advanced cancer for annual and biennial screeners in the BCSC [7]. The model includes age, race and ethnicity, BI-RADS breast density, BMI, first-degree family history of breast cancer, menopausal status and history of breast biopsy and result (https://tools.bcsc-scc.ucdavis.edu/AdvBC6yearRisk/#/). Postmenopausal obese women with heterogeneously or extremely dense breasts undergoing annual or biennial screening are at intermediate to high advanced cancer risk and premenopausal obese women with extremely dense breasts undergoing annual or biennial screening are at intermediate advanced cancer risk. Since premenopausal and postmenopausal women with calcifications, dense breasts, and BMI ≥ 25 kg/m^2^ have an advanced cancer risk over fivefold higher than among women with BMI < 25 kg/m^2^ without calcifications and non-dense breasts, including calcifications in the advanced cancer risk model could help identify women at very high advanced cancer risk who may benefit from more frequent screening and/or supplemental screening with ultrasound or magnetic resonance imaging (MRI). Targeting women at highest advanced cancer risk who may benefit from supplemental imaging could maximize potential benefits and minimize the harms of supplemental imaging [8, 38] and identify women who may benefit from weight reduction. Future studies should examine associations of types of calcifications with advanced cancer risk.

Our study was based on radiologists’ reports of calcifications and breast density on mammograms, relying on their experience and application of the BI-RADS manual in clinical practice. Thus, the prevalence of mammographic calcifications and association with cancer reflect clinical practice and radiologists’ preference to report calcifications. As a result, our results may underestimate prevalence and under or overestimate risk depending on the type of calcifications radiologists report. We analyzed data from film, digital and tomosynthesis breast images. Given mammographic calcifications are easily identified on breast imaging, it is unlikely that imaging modality type influenced risk estimates. We started follow-up three months after the screening mammogram to minimize including cancers directly associated with calcifications in the analysis. Given the calcification association with cancer was similar for mammograms with an initial BI-RADS assessment of 1 or 2 vs. 0 (and negative/benign final assessment) and the associations did not vary over the 5-year follow-up period, inclusion of false-negative cancers that present at screening but are diagnosed more than 3 months later is unlikely to have impacted the risk associations. Type of mammographic calcification was not available. We infer radiologists were identifying benign calcifications (e.g., coarse, rod-like, round, dystrophic, skin, vascular) when assigning a BI-RADS 1 or 2 assessment. We adjusted models for variables known to be strongly associated with advanced cancer risk. We did not adjust for reproductive factors which could confound or modify the association with mammographic calcifications. Lastly, if some of the missing BMI depends on BMI itself, it could lead to misclassification of imputed BMI from the multiple imputation models, potentially leading to biased estimates of associations. However, given the vast majority of missing BMI is likely missing at random, we suspect any bias to be small.

## Conclusion

Calcifications identified by radiologists on screening mammography interpretation in clinical practice were associated with an increased risk of advanced invasive cancer and non-advanced invasive cancer. In both premenopausal and postmenopausal women, the presence of calcifications increased advanced cancer risk beyond being overweight or obese and having dense breasts. As with breast density, calcifications noted in mammography reports could be incorporated into risk models for use by primary care providers in estimating advanced cancer risk [7]. Future research should investigate the type of calcification most strongly associated with advanced cancer risk, whether discrimination improves when including mammographic calcifications in advanced cancer risk models, and whether AI algorithms that predict advanced cancers detect-specific types of calcifications [9, 39].

## Supplementary Information

Below is the link to the electronic supplementary material.

Figure 1 Study population. Supplementary file1 (DOCX 22 KB)

Figure 2 Unadjusted cumulative incidence function for non-advanced invasive cancer in pre-menopausal women. Supplementary file2 (PNG 316 KB)

Figure 3 Unadjusted cumulative incidence function for non-advanced invasive cancer in post-menopausal women. Supplementary file3 (PNG 331 KB)

Supplementary file4 (DOCX 24 KB)

## Data Availability

The de-identified dataset underlying this manuscript will be shared upon email request to the BCSC Statistical Coordinating Center (kpwa.scc@kp.org). Some variables in the dataset may require approval from state agencies to allow third-party data sharing.
