# Supplementary figures and images for: Mammographic calcifications association with risk of advanced breast cancer
Source: Breast Cancer Res Treat. 2025 Jun 17;212(3):555–67. doi: 10.1007/s10549-025-07753-z (PMC12209027; doi:10.1007/s10549-025-07753-z)

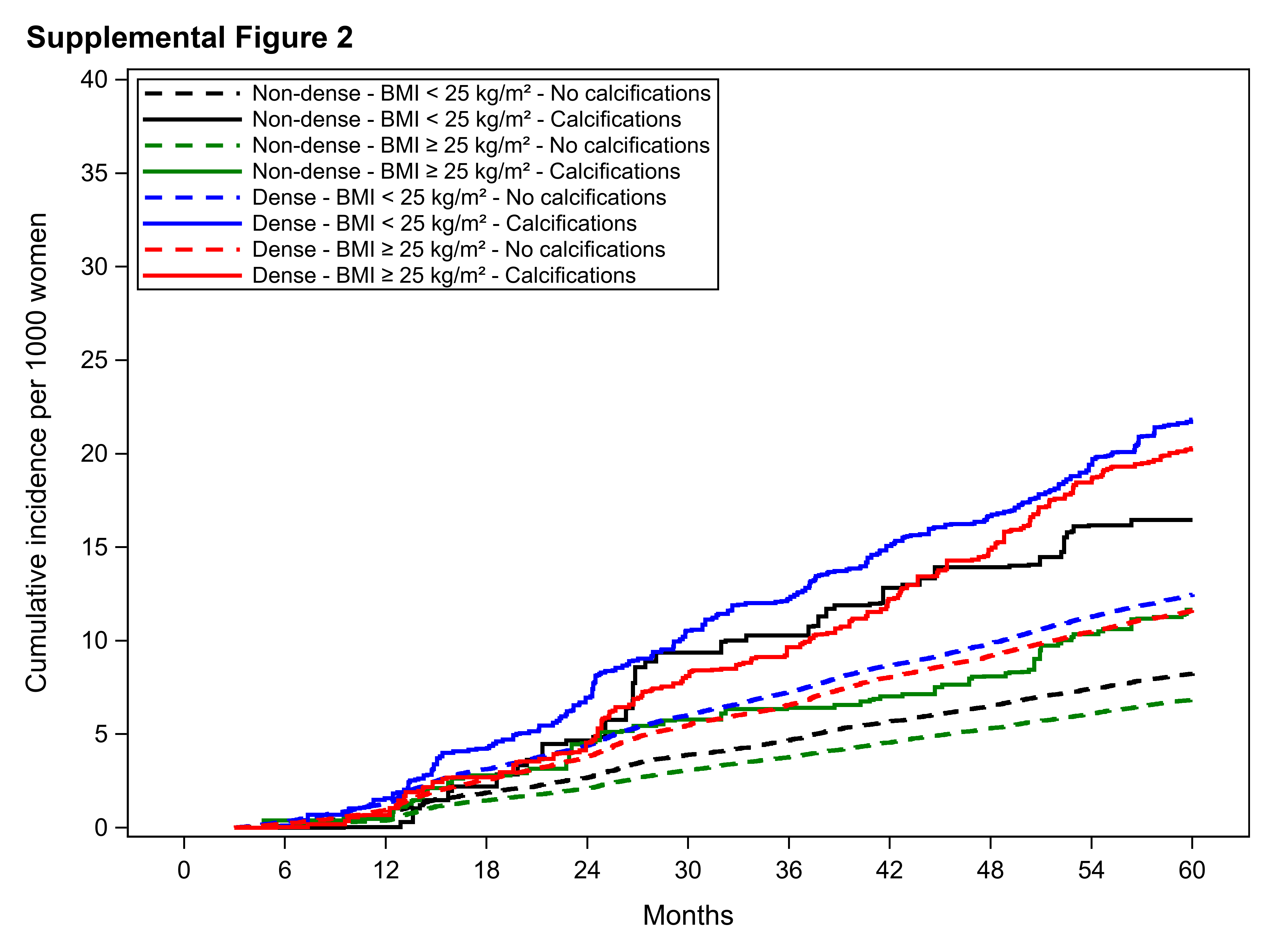

Supplement: Supplementary file 2 — Figure 2 Unadjusted cumulative incidence function for non-advanced invasive cancer in pre-menopausal women. Supplementary file2 (PNG 316 KB) [file 10549_2025_7753_MOESM2_ESM.png]

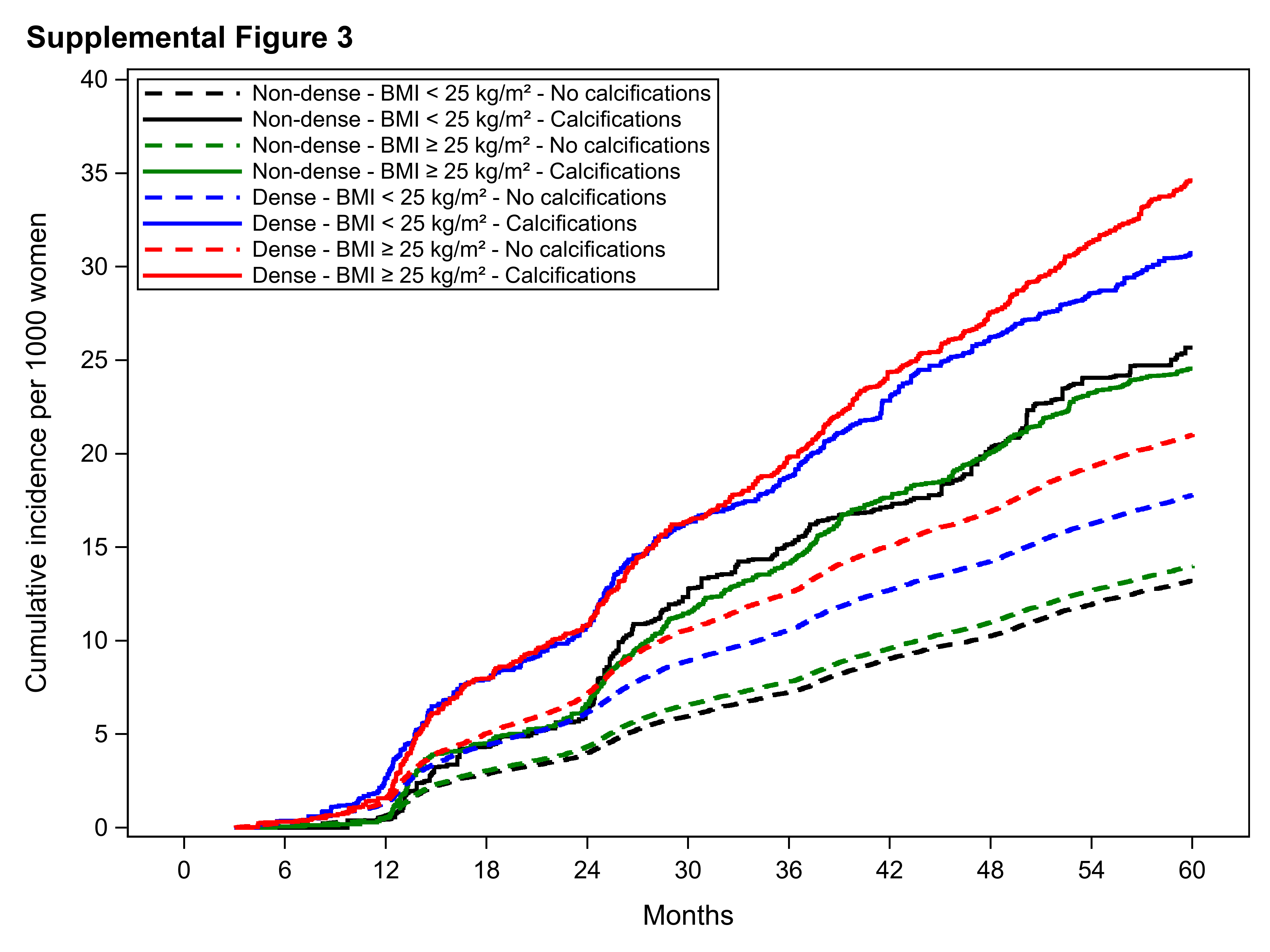

Supplement: Supplementary file 3 — Figure 3 Unadjusted cumulative incidence function for non-advanced invasive cancer in post-menopausal women. Supplementary file3 (PNG 331 KB) [file 10549_2025_7753_MOESM3_ESM.png]
