# Supplementary material for: Mammographic calcifications association with risk of advanced breast cancer
Source: Breast Cancer Res Treat. 2025 Jun 17;212(3):555–67. doi: 10.1007/s10549-025-07753-z (PMC12209027; doi:10.1007/s10549-025-07753-z)
Supplement: Supplementary file 4 — Supplementary file4 (DOCX 24 KB) [file 10549_2025_7753_MOESM4_ESM.docx]

**Supplemental Table 1**. Prevalence of calcifications by body mass index, menopausal status, and breast density^a^

| **BMI kg/m^2^** | | **Menopausal status** | **Non-dense**  **% (95% CI)** | | **Dense**  **% (95% CI)** |
| --- | --- | --- | --- | --- | --- |
| BMI < 25 | Premenopausal | | 1.4 (1.4, 1.5) | | 2.9 (2.8, 2.9) |
| BMI < 25 | Postmenopausal | | 2.8 (2.8, 2.9) | | 4.6 (4.5, 4.6) |
| BMI >25 | Premenopausal | | 1.9 (1.8, 1.9) | | 3.2 (3.1, 3.2) |
| BMI >25 | Postmenopausal | | 4.1 (4.1, 4.1) | | 6.0 (6.0, 6.1) |
|  | |  | |  |  |
| ^a^Average percentage over 45 imputed datasets  Non-dense (almost entirely fatty and scattered fibroglandular density); Dense (heterogeneously and extremely dense breast density); body mass index (BMI) | | | | | |

**Supplemental Table 2.** Multivariable hazard ratios for risk of advanced and non-advanced breast cancer associated with the presence of calcifications, menopausal status, body mass index, and breast density.

| **Clinical and imaging risk factors** | **Advanced cancer^a^** | **Non-advanced cancer^b^** |
| --- | --- | --- |
|  | **HR (95% CI)^c^** | **HR (95% CI) ^c^** |
| **Mammography calcifications** |  |  |
| No | ref | ref |
| Yes | 1.4 (1.3,1.7) | 1.4 (1.4,1.5) |
| **Menopausal** |  |  |
| No | ref | ref |
| Yes | 1.0 (0.9,1.1) | 1.0 (0.9,1.0) |
| **Body mass index, kg/m^2^** |  |  |
| Underweight/Normal (<25.0) | ref | ref |
| Overweight (25.0-29.9) | 1.5 (1.4,1.7) | 1.1 (1.1,1.2) |
| Obese I-III (>=30.0) | 2.0 (1.8,2.2) | 1.2 (1.1,1.2) |
| **BI-RADS breast density** |  |  |
| Almost entirely fat | ref | ref |
| Scattered fibroglandular densities | 2.7 (2.2,3.3) | 1.9 (1.7,2.0) |
| Heterogeneously dense | 5.5 (4.5,6.7) | 2.6 (2.4,2.8) |
| Extremely dense | 7.7 (6.2,9.6) | 3.0 (2.8,3.3) |

^a^Invasive cancer American Joint Committee on Cancer (AJCC) 8^th^ edition prognostic pathologic stage II or higher

^b^Invasive cancer AJCC 8^th^ edition prognostic pathologic stage I

^c^Hazard ratios are based on models that included presence of calcifications, menopausal status, BMI, breast density, adjusted for age (quadratic), race/ethnicity, 1st degree breast cancer family history, history of benign biopsy, time since last mammogram, and stratified by mammography registry. One model was fit for each outcome, while treating the other tumor type and DCIS as competing risks.

**Supplemental Table 3.** Comparing risk of advanced cancer associated with the presence of calcifications over time

| **Years since screening exam** | **HR (95% CI)** |
| --- | --- |
| 1-3 years vs within 1 year | 1.2 (0.7, 1.9) |
| 3-5 years vs within 1 year | 1.0 (0.6, 1.7) |
| 3-5 years vs 1-3 years | 0.9 (0.6, 1.3) |
